# Pathology-based thermal ablation safety margin for follicular adenoma: a digital whole-slide study of follicular carcinoma invasion

**DOI:** 10.3389/fendo.2026.1826810

**Published:** 2026-05-21

**Authors:** Yunfeng Qiao, Yun Niu, Zhenlong Zhao, Hanxiao Zhao, Yuton Liu, Ming-an Yu

**Affiliations:** 1 Beijing University of Chinese Medicine, Beijing, China; 2 Department of Interventional Medicine, China-Japan Friendship Hospital, Beijing, China; 3 Department of Pathology, China-Japan Friendship Hospital, Beijing, China

**Keywords:** ablation safety margin, capsular infiltration, digital pathology, Follicular adenoma, Follicular thyroid carcinoma, minimally infiltration, Thermal ablation

## Abstract

**Background:**

Follicular adenoma (FA) and follicular thyroid carcinoma (FC) are difficult to differentiate before surgery, raising concerns that thermal ablation (TA) of FA might treat an occult minimally invasive FC. Therefore, the microscopic extent of FC invasion was quantified to propose a pathology-informed ablation safety margin.

**Methods:**

A retrospective analysis was conducted on 43 surgically resected FCs treated between 2016 and 2023. Two readers independently reviewed the pathological images (×40 magnification). The capsule infiltration distance (the maximum depth of infiltration beyond the capsule) and the minimally invasive infiltration distance (the ultrasound-undetectable occult infiltration depth) were measured. These distances were compared by tumor stage, FC pathological classification, and lymph node metastasis (LNM).

**Results:**

The capsule infiltration distance ranged from 0.14 to 6.10 mm (mean, 1.49 ± 1.22). The minimally invasive infiltration distance ranged from 0.09 to 1.36 mm (mean, 0.56 ± 0.37). The distances did not differ by tumor stage, FC pathological classification, sex, or lymph node metastasis (LNM) (all P > 0.05). Age was associated with a larger capsule infiltration distance (P = 0.040). However, age was not associated with the minimally invasive infiltration distance (P = 0.367).

**Conclusion:**

The minimally invasive infiltration in FC did not exceed 1.36mm in this cohort. Since the actual ablation area achieved by clinical TA exceeds the pathological margin, this supports the extended ablation safety margin of 2mm beyond the sonographic tumor boundary. This TA margin could cover occult minimally invasive FC.

## Introduction

Thyroid cancer is the most common malignant tumor of the endocrine system (1). More than 90% of thyroid cancers come from differentiated follicular epithelial cells. Among them, follicular thyroid carcinoma (FC) accounts for about 10–15% (2–5).

Both follicular adenoma (FA) and follicular carcinoma (FC) originate from follicular cells. In pathology, a tumor is classified as FA when it shows capsular infiltration but does not cross the capsule, whereas it is classified as FC when the tumor crosses the capsule or extends into nearby thyroid tissue (6). In other words, FC exhibits infiltration beyond the capsule, while FA does not. However, fine needle aspiration (FNA) or core needle biopsy (CNB) cannot reliably differentiate between benign and malignant follicular tumors because they cannot assess full capsule invasion. In many cases, the final diagnosis requires a complete review of the tumor capsule after surgery. However, thyroid surgery can lead to complications such as hypothyroidism and hypoparathyroidism (7, 8).

Thermal ablation (TA) is safe and effective for benign and malignant thyroid nodules (9–12), and several studies have focused on TA for FA (13, 14). However, TA has not been widely used for FA because some occult minimally infiltrated FC may also be treated. This occurs because minimal infiltration is typically not visible on preoperative imaging, such as high-frequency ultrasound.

Therefore, the microscopic extent to which minimally invasive FC may extend beyond the capsule needs to be defined. This information can help inform an ablation safety margin that is sufficient to encompass ultrasound-occult minimal invasion during TA for FA. Consequently, the aim of the present study is to measure the invasion distance in FC and evaluated associated risk factors.

## Materials and methods

### Patients

This retrospective study was approved by the Human Ethics Review Committee of the China-Japan Friendship Hospital. Medical records were reviewed for patients with FC who underwent surgical resection between October 2016 and October 2023. Because all personal information was kept confidential, informed consent for inclusion in this retrospective study was waived.

### Inclusion and exclusion criteria

The inclusion criteria were: (1) patients who underwent thyroid lobectomy or total thyroidectomy, and (2) postoperative pathology confirming FC.

The exclusion criteria were: (1) unavailable pathology slides or digital images; (2) Diffuse capsular invasion hindering infiltration measurement; and (3) less than 2 mm of normal thyroid tissue surrounding the tumor on digitized slides.

**Figure 1:**
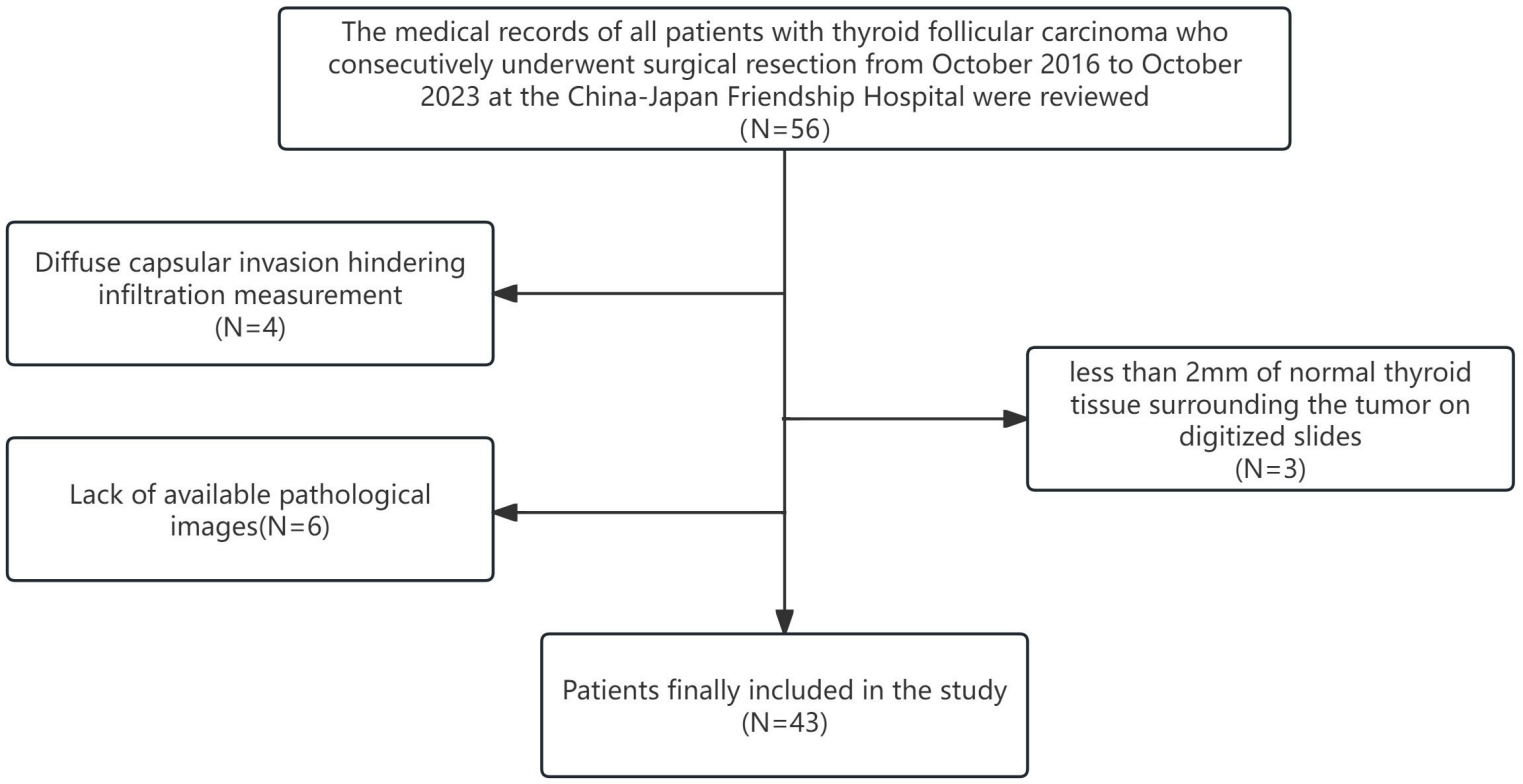
Study flowchart.

The patient selection process is shown in **Figure 1**.

### Grouping

FC cases were grouped according to pathological type:

1. Minimally invasive type (Type I): Tumor cells invade beyond the capsule and into thyroid tissue, but the invasion is limited to the thyroid capsule.
2. Encapsulated angioinvasive type (Type II): Vascular invasion is present, with or without capsular invasion. Tumor cells invade blood vessels, and tumor thrombi can be seen in intracapsular vessels.
3. Widely invasive type (Type III): The tumor breaks through the thyroid capsule at multiple sites and invades a large area, including extra-thyroid tissue.

Cases were also grouped by maximum tumor diameter (tumor stage):

1. T1 of FC: Tumor ≤ 2 cm and limited to the thyroid.
2. T2 of FC: Tumor > 2 cm and ≤ 4 cm, limited to the thyroid.
3. T3 of FC: Tumor > 4 cm, classified based solely on maximum tumor diameter.

For the purposes of this study, tumor stage grouping was determined by maximum tumor diameter; At the same time, no patients with strap muscle invasion were identified in the present cohort.

This grouping method was based on the final pathology report according to 2022 WHO Classification of Neuroendocrine Neoplasms (15).

### Image acquisition for digital pathology

Thyroid specimens obtained from unilateral or bilateral lobectomy were analyzed. After resection, tissues were fixed in 10% formaldehyde, sectioned at 4 μm, and stained with hematoxylin and eosin (H&E). In cases of multifocal FC, the largest nodule was selected as the dominant nodule. For very large tumors requiring subdivision, all sections demonstrating the maximum tumor diameter were selected and defined as the target slides. All target slides were scanned as whole-slide images (WSI) at ×40 magnification.

### Digital image analysis

To improve accuracy, two authors (Y.F. Qiao and Y. Niu) underwent standardized training on capsular infiltration measurement criteria before the commencement of the study. Both independently analyzed all digitized slides using Image Viewer (Shengqiang Technology). Discrepancies were resolved through joint review of the slides until consensus was achieved.

**Figure 2:**
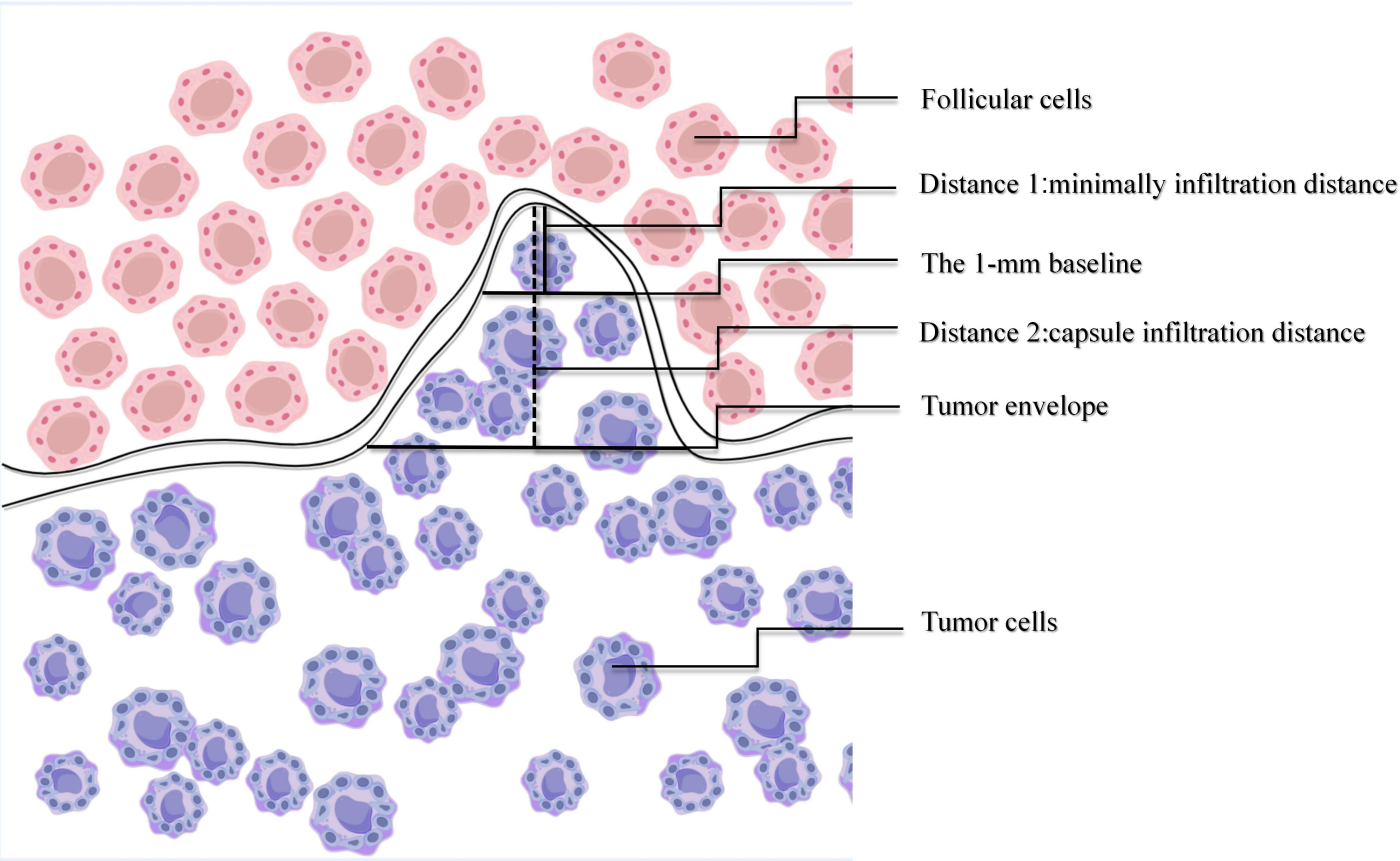
Diagram of measurement standards: Measuring capsule infiltration distance: the vertical distance from the tumor capsule to the farthest point of invasion (the horizontal black solid line indicates the tumor capsule; the vertical red solid line indicates Distance 2: the measured vertical distance). Measuring minimal invasion distance: the vertical distance from the farthest point of tumor invasion to a 1mm wide base (the horizontal black short solid line indicates the 1 mm wide base; the vertical green solid line indicates Distance 1: the measured distance).

**Figure 3:**
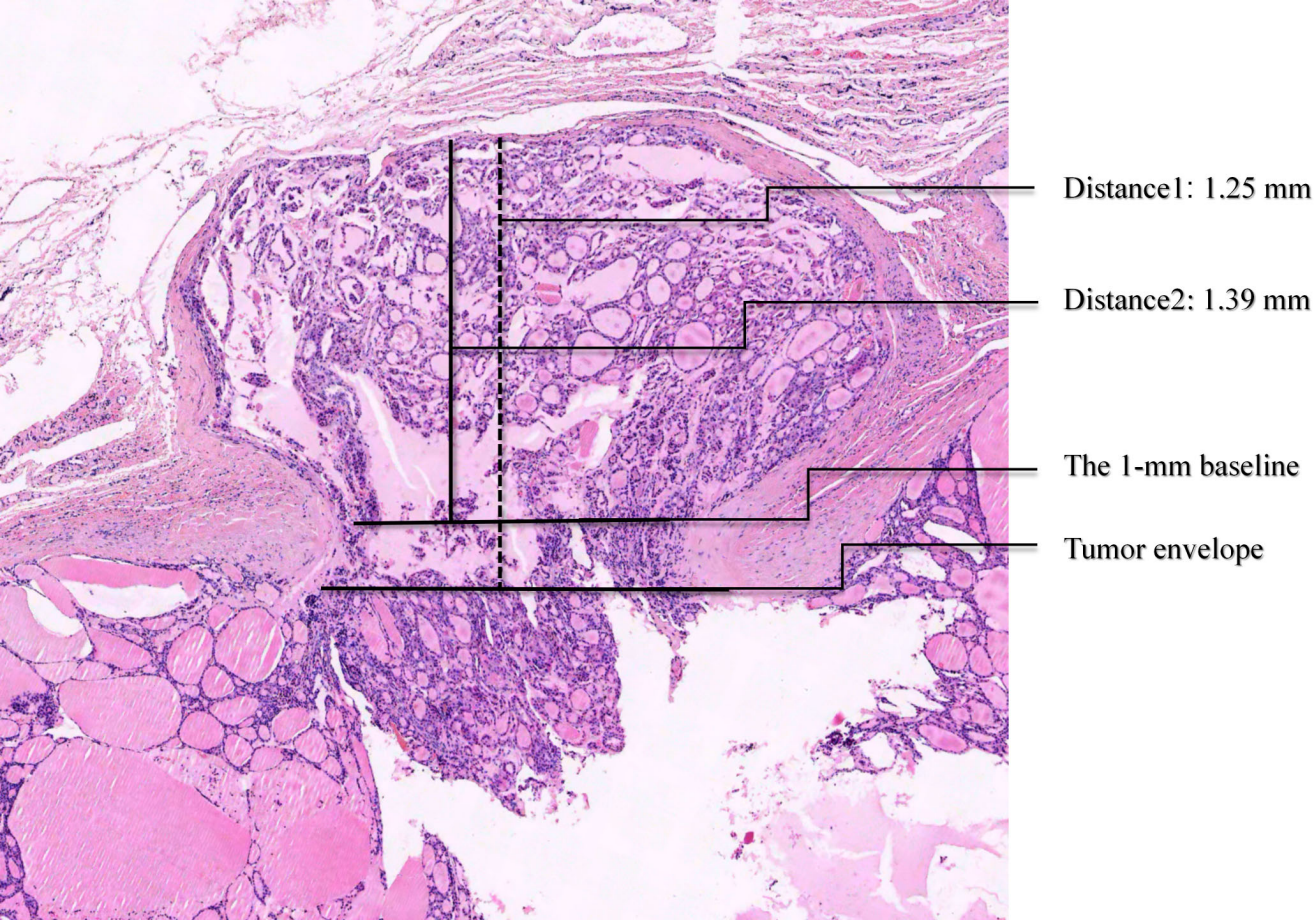
23-year-old female patient with a thyroid follicular tumor (FTC). The largest nodule (0.56 cm × 0.23 cm) is located in the right lobe of the thyroid, and the pathological type is Micro-invasive type (Type I). Measurement of capsule infiltration distance: the vertical distance from the tumor capsule to the farthest invasion point is 1.39 mm (horizontal white solid line indicates the tumor capsule; vertical white solid line indicates Distance 2: measurement distance). Measurement of minimal invasion distance: the vertical distance from the farthest invading point of the tumor to a 1 mm wide base is 1.25 mm (horizontal yellow solid line indicates the 1 mm wide base; vertical yellow solid line indicates Distance 1: measurement distance). (H&E, ×40).

The capsular infiltration distance was defined as the vertical distance from the tumor capsule to the deepest point of invasion (**Figures 2**, **3**).

The minimal infiltration distance was defined with reference to the resolution of high-frequency ultrasound. In clinical practice, invasion with a width <1 mm is typically undetectable on high-frequency ultrasound(7.5–15 MHz) (16, 17). Accordingly, the distance was measured from the farthest point of infiltration to the location at which the infiltration width reached 1 mm (**Figures 2**, **3**).

### Statistical analysis

SPSS (Version 26.0) was used for statistical analysis. For descriptive statistics, variables that did not follow a normal distribution were described using the median and interquartile ranges (IQR), while categorical variables were expressed as percentages. To compare infiltration distances between different groups, a rank-sum test and pairwise rank-sum tests were performed, with the Bonferroni correction applied for multiple comparisons within groups. Rank correlation analysis was used to evaluate the correlation between the maximum diameter of FC and the infiltration distance. Multiple linear regression was employed to analyze the risk factors associated with the infiltration distance. A P value of less than 0.05 was considered statistically significant.

## Results

### Patient characteristics

A total of 43 patients who underwent surgical resection of FC were included in this study, of which 17/43 (39.5%) were males and 26/43 (60.5%) were females. The median age was 51 years (IQR, 30–58 years). Based on the maximum diameter of the tumor, the cases were divided into three stages: 17/43 (39.5%) were classified as T1 FCs, 15/43 (34.9%) as T2 FCs, and 11/43 (25.6%) as T3 FCs. According to the pathological classification of FC, the cases were divided into three groups: 18/43 (41.9%) of type I, 8/43 (18.6%) of type II, and 17/43 (39.5%) of type III. Regarding LNM analysis, a total of 12/43 (27.9%) patients had LNM, of which 8/12 (66.7%) were type I, 2/12 (16.7%) were type II, and 2/12 (16.7%) were type III, while the remaining patients had no LNM (**Table 1**).

**Table 1:** Baseline patient characteristics.

Characteristics	Data
Age (years)	51 (30-58)
Sex	
Female	26 (60.5%)
Male	17 (39.5%)
Tumor stage	
T1(≤2cm)	17 (39.5%)
T2 (>2cm and ≤4cm)	15 (34.9%)
T3(>4cm)	11 (25.6%)
Maximum tumor diameter, cm	2.3 (1.5-4-5)
Lymph node metastasis	
Present	12 (27.9%)
Absent	31 (72.1%)
Follicular cancer classification	
Micro-infiltrating type (Type I)	18(41.9%)
Encapsulated vascular infiltrating type (Type II)	8 (18.6%)
Extensively infiltrated type (Type III)	17 (39.5%)

Data are presented as the median (interquartile range) or number of patients (percentages).

### Capsule infiltration distance

Of these 43 patients, the capsule infiltration distance ranged from 0.14 mm to 6.10 mm (mean: 1.49 ± 1.22 mm). For subgroup analysis based on the maximum tumor diameter, the median capsule infiltration distance was 1.86 mm (range: 0.25-6.10 mm; IQR, 1.06-2.66 mm) for T1 FCs, 1.32 mm (range: 0.14-3.37 mm; IQR, 0.77-1.86 mm) for T2 FCs, and 1.14 mm (range: 0.30-2.86 mm; IQR, 0.63-1.66 mm) for T3 FCs. There was no significant difference in capsule infiltration distance between the three groups (P = 0.408).

In terms of pathological classification, the median capsule infiltration distance was 1.59 mm (range: 0.25-6.10 mm; IQR, 0.91-2.27 mm) for type I, 1.52 mm (range: 0.31-4.41 mm; IQR, 1.03-2.01 mm) for type II, and 0.58 mm (range: 0.14-1.30 mm; IQR, 0.22-0.80 mm) for type III. No significant difference in capsule infiltration distance was observed between the three types (P = 0.210).

Regarding lymph node metastasis (LNM), the capsule infiltration distance was 1.17 mm (range: 0.14-3.01 mm) for FC with LNM and 1.61 mm (range: 0.25-6.10 mm) for FC without LNM. There was no significant difference in capsule infiltration distance between these two groups (P = 0.372).

### Minimally infiltration distance

Of these 43 patients, the minimally infiltration distance ranged from 0.09 mm to 1.36 mm (mean: 0.56 ± 0.37 mm). When grouped by maximum tumor diameter, the median minimally infiltration distance was 0.58 mm (range: 0.17-1.35 mm; IQR, 0.37-0.79 mm) for T1 FCs, 0.51 mm (range: 0.09-1.36 mm; IQR, 0.31-0.71 mm) for T2 FCs, and 0.59 mm (range: 0.17-1.20 mm; IQR, 0.37-0.81 mm) for T3 FCs. There was no significant difference in minimally infiltration distance between the three groups (P = 0.828).

Concerning pathological classification, the median minimally infiltration distance was 0.56 mm (range: 0.17-1.35 mm; IQR, 0.39-0.73 mm) for type I, 0.58 mm (range: 0.09-1.36 mm; IQR, 0.40-0.76 mm) for type II, and 0.39 mm (range: 0.14-0.83 mm; IQR, 0.17-0.52 mm) for type III. No significant difference in minimally infiltration distance was observed between the three types (P = 0.595).

As for LNM, the minimally infiltration distance was 0.52 mm (range: 0.09-1.35 mm) for FC with LNM and 0.58 mm (range: 0.17-1.36 mm) for FC without LNM. There was no significant difference in minimally infiltration distance between the two groups (P = 0.456) (**Table 2**).

**Table 2:** Univariate analysis of risk factors for capsule infiltration distance and minimally infiltration distance.

Characteristics	Capsule infiltration distance P value	Minimally infiltration distance P value
Age	0.040^c*^	0.367^c^
Sex	0.157^c^	0.477^c^
Maximum tumor diameter, cm	0.258^a^	0.822^a^
Tumor stage	0.408^b^	0.828^b^
FC typing	0.210^a^	0.595^a^
Lymph node metastasis	0.372^b^	0.456^b^
Multiple comparisons		
Type I vs. Type II	0.848	0.848
Type I vs. Type III	0.189	0.189
Type II vs. Type III	0.220	0.220

The statistical tests used were as follows: ^a^Mann-Whitney U test; ^b^Kruskal-Wallis H test; and ^c^Spearman’s rank correlation. *Statistically significant difference (P<0.05).

### Influencing factors

According to the results of univariate and multivariate analysis, capsule infiltration distance and minimally infiltration distance were not associated with sex, tumor stage, maximum tumor diameter, LNM, or FC pathological classification. However, capsule infiltration distance was associated with age (P = 0.036). In multivariable linear regression, older age remained significantly correlated with capsule infiltration distance (P = 0.040). In contrast, age was not associated with minimally infiltration distance (P = 0.367) (**Table 2**).

## Discussion

Several recent clinical studies have explored TA for thyroid follicular neoplasm (FN). One study reported that radiofrequency ablation (RFA) of 10 small (<2 cm) FN led to a mean volume reduction of about 99% and complete disappearance in 80% of lesions. It also found no local recurrence during a mean follow-up of more than 5 years. This suggests that TA can be a safe and effective option for patients who refuse surgery (18, 19). More recently, studies also reported TA in broader FN groups, which also showed low disease progression rate and complications (13, 18, 20–22). However, the available evidence primarily focuses on imaging endpoints, such as volume reduction, complete disappearance, and disease progression, and provides limited pathologic detail. Key histopathologic parameters—including capsular infiltration distance, minimally infiltration distance, and the ablation safety margin required to encompass microscopic invasion—are generally not reported. The present study extends the literature by quantifying capsular infiltration distance and minimal infiltration distance in FC. Based on these measurements, a pathology-informed safety margin is proposed for TA when treating FA, which may include occult minimally infiltrated FC.

Invasion into surrounding normal tissue is a key pathological sign of malignancy in FN. Even with better ultrasound, tumors with the minimally infiltration distance is still hard to detect. This is because ultrasound has limited spatial resolution, especially when the infiltration distance is <1 mm. Recent digital pathology studies have shown that for the ablation treatment of papillary thyroid carcinoma, it is recommended to extend the ablation range by 2.5 millimeters beyond the visible tumor boundary to effectively reduce the risk of local recurrence of PTC after thermal ablation (23–25).

However, when TA is used for FA, minimally invasive FC cannot be completely excluded, and some cases may be inadvertently treated. The results in present study showed that maximum capsular invasion distance was 6.10 mm, while the median minimally infiltration distance was only 0.56 mm (ranged from 0.09 mm to 1.36 mm), which indicate that the extended ablation safety margin of 2 mm beyond the sonographic tumor boundary is sufficient to encompass the microscopic invasion at the pathological level. Moreover, there were no significant differences in either capsular invasion distance or minimally infiltration distance across tumor stage, FC pathological classification, or LNM status (all P>0.05). Only age was related to capsular invasion distance (P = 0.040), but not related to minimally infiltration distance (P = 0.367). It is also reported in prior studies that age is an important risk factors for aggressiveness and prognosis in FC (10). However, the maximum minimally infiltration distance was 1.36 mm despite the old age, which confirms that the extended ablation safety margin of 2 mm is sufficient.

In clinical practice, the main goal of extended ablation is to cover minimally infiltration distance, because it is not visible on ultrasound. Based on the results of the present study, minimally infiltration distance was not related to FC pathological classification, maximum tumor diameter, or LNM, and a uniform extension of >2 mm may be a reasonable and a choice for TA in this setting, regardless of these factors. The FC data are derived from quantified microscopic invasion boundaries and add pathologic evidence to current TA practice (26–28).

Several limitations should be noted. First, it is a retrospective study and is therefore subject to inherent selection bias. Second, it was conducted in a single center with a relatively small sample size, which may limit the generalizability of the findings. Further studies with larger, preferably multicenter cohorts are needed to confirm these results, to explore whether different FC variants require different margins, and to integrate vascular invasion and other prognostic factors into a more comprehensive ablation strategy.

In conclusion, the present study indicates that the minimally infiltration distance of FC is less than 1.36 mm in all evaluated cases. An extended ablation safety margin of approximately >2 mm could be sufficient to achieve complete ablation not only for FA, but also for FC that may be inadvertently included. The findings provide the first quantitative, FC-specific evidence that links histopathological infiltration distance to a practical ablation safety margin and refine existing recommendations for TA of FN.

## Data Availability

The datasets presented in this article are not readily available due to patient privacy and confidentiality constraints. Requests for data access should be directed to the corresponding author and are subject to review by the institutional ethics committee.
